# Bis[4-(2-hydr­oxy-3-methoxy­benzyl­ideneamino)phen­yl] ether

**DOI:** 10.1107/S1600536808014980

**Published:** 2008-05-24

**Authors:** Hong-Wu Xu, Jin-Xia Li, Yin-Hua Li

**Affiliations:** a Department of Materials and Chemical Engineering, ZhongYuan University of Technology, Zhengzhou, Henan 450007, People’s Republic of China

## Abstract

The title compound, C_28_H_24_N_2_O_5_, a flexible Schiff base ligand, was prepared in high yield by a Schiff base condensation of 3-methoxy­salicylaldehyde and bis­(4-amino­phen­yl) ether in methanol. The mol­ecule lies on a twofold rotation axis, and each half exhibits an imine *E* configuration and an O—H⋯N hydrogen bond. The dihedral angle between the two benzene rings attached to the central O atom is 69.22 (6)°, and that between each of these rings and the other benzene ring in the same half of the mol­ecule is 24.29 (11)°, illustrating the degree of twisting of the flexible mol­ecule.

## Related literature

For related literature, see: Chu *et al.* (2007 ▶); Guo *et al.* (2002 ▶); He *et al.* (2000 ▶); Tesouro Vallina *et al.* (2001 ▶); Yoshida *et al.* (1999 ▶).
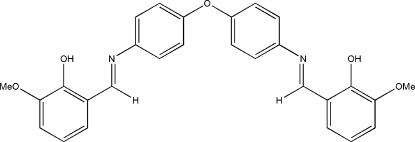


## Experimental

### 

#### Crystal data

C_28_H_24_N_2_O_5_
*M* *_r_* = 468.49
Monoclinic, 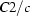

*a* = 15.585 (7) Å
*b* = 7.578 (4) Å
*c* = 19.859 (9) Å
β = 92.760 (8)°
*V* = 2342.7 (19) Å^3^
*Z* = 4
Mo *K*α radiation
μ = 0.09 mm^−1^
*T* = 173 (2) K
0.30 × 0.20 × 0.20 mm

#### Data collection

Bruker SMART CCD diffractometer
Absorption correction: none
8736 measured reflections
2689 independent reflections
2016 reflections with *I* > 2σ(*I*)
*R*_int_ = 0.035

#### Refinement

*R*[*F* ^2^ > 2σ(*F* ^2^)] = 0.061
*wR*(*F* ^2^) = 0.166
*S* = 1.11
2689 reflections
165 parameters
H atoms treated by a mixture of independent and constrained refinement
Δρ_max_ = 0.15 e Å^−3^
Δρ_min_ = −0.14 e Å^−3^

### 

Data collection: *SMART* (Bruker, 2001 ▶); cell refinement: *SAINT* (Bruker, 2001 ▶); data reduction: *SAINT*; program(s) used to solve structure: *SHELXS97* (Sheldrick, 2008 ▶); program(s) used to refine structure: *SHELXL97* (Sheldrick, 2008 ▶); molecular graphics: *SHELXTL* (Sheldrick, 2008 ▶); software used to prepare material for publication: *SHELXTL*.

## Supplementary Material

Crystal structure: contains datablocks I, global. DOI: 10.1107/S1600536808014980/cf2200sup1.cif

Structure factors: contains datablocks I. DOI: 10.1107/S1600536808014980/cf2200Isup2.hkl

Additional supplementary materials:  crystallographic information; 3D view; checkCIF report

## Figures and Tables

**Table 1:** Hydrogen-bond geometry (Å, °)

*D*—H⋯*A*	*D*—H	H⋯*A*	*D*⋯*A*	*D*—H⋯*A*
O2—H3⋯N1	0.84	1.87	2.611 (2)	147

**Fig. 1.**
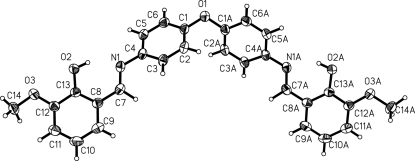
The molecular structure of the title compound with the atomic numbering scheme. Displacement ellipsoids are drawn at the 30% probability level. [Symmetry code A: 2-x, y, 3/2-z.]

## supplementary crystallographic information

### Comment

Within the field of supramolecular inorganic chemistry, self-assembly is one of the most efficient methods for complex architectures comprising spatially and geometrically well defined arrays of metal ions. Because of easy syntheses by simple one-pot condensation reactions between aldehydes (or ketones) and amines and their coordinating ability with metal ions, multidentate Schiff base ligands such as pyridylimines (He *et al.*, 2000; Guo *et al.*, 2002; Tesouro Vallina *et al.*, 2001) and salicyladimines (Yoshida *et al.*, 1999; Chu *et al.*, 2007) were designed and used to prepare complexes in recent years. Here we report the synthesis and structure of a new flexible Schiff base ligand, bis(*N*-(3-methoxysalicylidene)-4-aminophenyl) ether. The molecule lies on a twofold rotation axis, and each half exhibits an imine E configuration and an O—H···N hydrogen bond. The dihedral angle between the two benzene rings attached to the central O atom is 69.22 (6)°, and that between each of these rings and the other benzene ring in the same half of the molecule is 24.29 (11)°, illustrating the degree of twisting of the flexible molecule. The bond lengths and angles are in agreement with those reported for other salicyladimines ligands (Chu *et al.*, 2007).

### Experimental

The title compound was prepared by a Schiff-base condensation of 3-methoxysalicylaldehyde (3.04 g, 20 mmol) and bis(4-aminophenyl) ether (2.02 g, 10 mmol) in methanol (40 ml). The solution was stirred and refluxed for 1 day. The orange precipitate was filtered off, washed with a small amount of methanol and dried *in vacuo*. Yield: 91%. Well shaped orange crystals suitable for X-ray diffraction analysis were grown by slow evaporation of a chloroform solution of the title compound at room temperature.

### Refinement

H atoms were positioned geometrically (C—H = 0.96 Å; O—H = 0.84 Å), assigned isotropic displacement parameters equal to 1.2U_eq_ of the parent atoms, and allowed to ride on these parent atoms.

### Crystal data

**Table:**

C_28_H_24_N_2_O_5_	*F*_000_ = 984
*M**_r_* = 468.49	*D*_x_ = 1.328 Mg m^−^^3^
Monoclinic, *C*2/*c*	Mo *K*α radiation λ = 0.71073 Å
*a* = 15.585 (7) Å	Cell parameters from 2635 reflections
*b* = 7.578 (4) Å	θ = 1.7–25.1º
*c* = 19.859 (9) Å	µ = 0.09 mm^−^^1^
β = 92.760 (8)º	*T* = 173 (2) K
*V* = 2342.7 (19) Å^3^	Prism, colourless
*Z* = 4	0.30 × 0.20 × 0.20 mm

### Data collection

**Table:**

Bruker SMART CCD diffractometer	2016 reflections with *I* > 2σ(*I*)
Radiation source: fine-focus sealed tube	*R*_int_ = 0.035
Monochromator: graphite	θ_max_ = 27.5º
*T* = 173(2) K	θ_min_ = 2.6º
ω scans	*h* = −20→19
Absorption correction: none	*k* = −9→9
8736 measured reflections	*l* = −22→25
2689 independent reflections	

### Refinement

**Table:**

Refinement on *F*^2^	Secondary atom site location: difference Fourier map
Least-squares matrix: full	Hydrogen site location: inferred from neighbouring sites
*R*[*F*^2^ > 2σ(*F*^2^)] = 0.062	H atoms treated by a mixture of independent and constrained refinement
*wR*(*F*^2^) = 0.166	*w* = 1/[σ^2^(*F*_o_^2^) + (0.0739*P*)^2^ + 0.5764*P*] where *P* = (*F*_o_^2^ + 2*F*_c_^2^)/3
*S* = 1.11	(Δ/σ)_max_ = 0.001
2689 reflections	Δρ_max_ = 0.15 e Å^−^^3^
165 parameters	Δρ_min_ = −0.13 e Å^−^^3^
Primary atom site location: structure-invariant direct methods	Extinction correction: none

### Special details

**Table:**

Geometry. All e.s.d.'s (except the e.s.d. in the dihedral angle between two l.s. planes) are estimated using the full covariance matrix. The cell e.s.d.'s are taken into account individually in the estimation of e.s.d.'s in distances, angles and torsion angles; correlations between e.s.d.'s in cell parameters are only used when they are defined by crystal symmetry. An approximate (isotropic) treatment of cell e.s.d.'s is used for estimating e.s.d.'s involving l.s. planes.
Refinement. Refinement of *F*^2^ against ALL reflections. The weighted *R*-factor *wR* and goodness of fit *S* are based on *F*^2^, conventional *R*-factors *R* are based on *F*, with *F* set to zero for negative *F*^2^. The threshold expression of *F*^2^ > σ(*F*^2^) is used only for calculating *R*-factors(gt) *etc*. and is not relevant to the choice of reflections for refinement. *R*-factors based on *F*^2^ are statistically about twice as large as those based on *F*, and *R*- factors based on ALL data will be even larger.

### Fractional atomic coordinates and isotropic or equivalent isotropic displacement parameters (Å^2^)

**Table:**

	*x*	*y*	*z*	*U*_iso_*/*U*_eq_	
N1	0.88064 (10)	0.0091 (2)	0.52377 (7)	0.0561 (4)	
O1	1.0000	−0.3717 (3)	0.7500	0.0694 (6)	
O2	0.78593 (9)	0.02484 (16)	0.41186 (7)	0.0660 (4)	
H3	0.8101	−0.0219	0.4461	0.079*	
C4	0.91503 (11)	−0.0853 (3)	0.58068 (8)	0.0527 (5)	
C13	0.80476 (12)	0.1980 (2)	0.41092 (9)	0.0521 (4)	
O3	0.71769 (10)	0.21180 (19)	0.31172 (8)	0.0739 (5)	
C12	0.77074 (13)	0.3000 (2)	0.35765 (10)	0.0572 (5)	
C3	0.98968 (12)	−0.0344 (3)	0.61733 (9)	0.0574 (5)	
H8	1.0210	0.0659	0.6036	0.069*	
C6	0.90099 (13)	−0.3309 (3)	0.65622 (10)	0.0610 (5)	
H9	0.8709	−0.4333	0.6694	0.073*	
C5	0.87355 (12)	−0.2368 (3)	0.59967 (10)	0.0591 (5)	
H10	0.8250	−0.2772	0.5733	0.071*	
C8	0.85726 (12)	0.2774 (2)	0.46152 (10)	0.0564 (5)	
C2	1.01805 (12)	−0.1291 (3)	0.67334 (9)	0.0581 (5)	
H12	1.0689	−0.0941	0.6981	0.070*	
C7	0.89139 (13)	0.1765 (3)	0.51873 (10)	0.0602 (5)	
C1	0.97295 (13)	−0.2738 (3)	0.69341 (9)	0.0562 (5)	
C11	0.79102 (15)	0.4744 (3)	0.35347 (12)	0.0718 (6)	
H15	0.7685	0.5426	0.3165	0.086*	
C14	0.66750 (17)	0.3156 (3)	0.26521 (12)	0.0825 (7)	
H16A	0.6388	0.4096	0.2895	0.124*	
H16B	0.6243	0.2408	0.2418	0.124*	
H16C	0.7048	0.3679	0.2322	0.124*	
C9	0.87598 (16)	0.4578 (3)	0.45639 (12)	0.0744 (6)	
H17	0.9110	0.5138	0.4905	0.089*	
C10	0.84410 (17)	0.5530 (3)	0.40260 (14)	0.0825 (7)	
H18	0.8584	0.6743	0.3988	0.099*	
H4	0.9250 (14)	0.249 (3)	0.5555 (12)	0.082 (7)*	

### Atomic displacement parameters (Å^2^)

**Table:**

	*U*^11^	*U*^22^	*U*^33^	*U*^12^	*U*^13^	*U*^23^
N1	0.0574 (9)	0.0669 (10)	0.0438 (8)	−0.0043 (8)	0.0010 (7)	−0.0009 (7)
O1	0.0892 (14)	0.0622 (12)	0.0554 (11)	0.000	−0.0097 (10)	0.000
O2	0.0793 (10)	0.0517 (7)	0.0653 (9)	−0.0103 (7)	−0.0143 (7)	0.0074 (6)
C4	0.0519 (10)	0.0664 (11)	0.0400 (9)	−0.0021 (8)	0.0041 (7)	−0.0068 (8)
C13	0.0562 (10)	0.0481 (10)	0.0528 (10)	−0.0019 (8)	0.0097 (8)	0.0000 (8)
O3	0.0880 (11)	0.0637 (9)	0.0679 (9)	0.0048 (7)	−0.0170 (8)	0.0087 (7)
C12	0.0614 (11)	0.0546 (11)	0.0561 (11)	0.0051 (9)	0.0081 (9)	0.0020 (9)
C3	0.0554 (10)	0.0723 (12)	0.0448 (10)	−0.0124 (9)	0.0057 (8)	−0.0027 (9)
C6	0.0654 (12)	0.0565 (11)	0.0610 (12)	−0.0098 (9)	0.0007 (10)	−0.0014 (9)
C5	0.0557 (11)	0.0639 (11)	0.0566 (11)	−0.0071 (9)	−0.0074 (9)	−0.0077 (9)
C8	0.0591 (11)	0.0536 (10)	0.0571 (11)	0.0004 (8)	0.0091 (9)	−0.0054 (8)
C2	0.0528 (10)	0.0767 (13)	0.0446 (10)	−0.0101 (9)	−0.0009 (8)	−0.0068 (9)
C7	0.0628 (12)	0.0658 (12)	0.0521 (11)	−0.0022 (10)	0.0030 (9)	−0.0116 (9)
C1	0.0614 (11)	0.0625 (11)	0.0443 (10)	0.0019 (9)	−0.0013 (8)	−0.0048 (8)
C11	0.0819 (15)	0.0571 (12)	0.0768 (14)	0.0050 (11)	0.0087 (12)	0.0074 (11)
C14	0.1016 (18)	0.0836 (15)	0.0610 (13)	0.0289 (13)	−0.0081 (12)	0.0036 (11)
C9	0.0833 (15)	0.0583 (12)	0.0814 (15)	−0.0083 (11)	0.0010 (12)	−0.0145 (11)
C10	0.1018 (18)	0.0466 (11)	0.0993 (19)	−0.0044 (12)	0.0076 (15)	0.0029 (12)

### Geometric parameters (Å, °)

**Table:**

N1—C7	1.284 (3)	C6—C1	1.382 (3)
N1—C4	1.421 (2)	C6—H9	0.950
O1—C1^i^	1.394 (2)	C5—H10	0.950
O1—C1	1.394 (2)	C8—C9	1.402 (3)
O2—C13	1.345 (2)	C8—C7	1.449 (3)
O2—H3	0.840	C2—C1	1.372 (3)
C4—C5	1.379 (3)	C2—H12	0.950
C4—C3	1.397 (3)	C7—H4	1.04 (2)
C13—C12	1.394 (3)	C11—C10	1.383 (3)
C13—C8	1.401 (3)	C11—H15	0.950
O3—C12	1.375 (2)	C14—H16A	0.980
O3—C14	1.419 (2)	C14—H16B	0.980
C12—C11	1.363 (3)	C14—H16C	0.980
C3—C2	1.379 (3)	C9—C10	1.363 (3)
C3—H8	0.950	C9—H17	0.950
C6—C5	1.381 (3)	C10—H18	0.950
			
C7—N1—C4	120.94 (16)	C1—C2—C3	120.11 (17)
C1^i^—O1—C1	115.8 (2)	C1—C2—H12	119.9
C13—O2—H3	109.5	C3—C2—H12	119.9
C5—C4—C3	118.51 (17)	N1—C7—C8	122.55 (17)
C5—C4—N1	118.23 (16)	N1—C7—H4	122.3 (12)
C3—C4—N1	123.24 (18)	C8—C7—H4	115.2 (12)
O2—C13—C12	118.43 (17)	C2—C1—C6	120.57 (17)
O2—C13—C8	121.98 (16)	C2—C1—O1	121.33 (16)
C12—C13—C8	119.59 (17)	C6—C1—O1	118.06 (18)
C12—O3—C14	117.23 (17)	C12—C11—C10	120.5 (2)
C11—C12—O3	124.41 (18)	C12—C11—H15	119.8
C11—C12—C13	120.21 (19)	C10—C11—H15	119.8
O3—C12—C13	115.39 (17)	O3—C14—H16A	109.5
C2—C3—C4	120.22 (18)	O3—C14—H16B	109.5
C2—C3—H8	119.9	H16A—C14—H16B	109.5
C4—C3—H8	119.9	O3—C14—H16C	109.5
C5—C6—C1	118.99 (19)	H16A—C14—H16C	109.5
C5—C6—H9	120.5	H16B—C14—H16C	109.5
C1—C6—H9	120.5	C10—C9—C8	120.2 (2)
C4—C5—C6	121.44 (17)	C10—C9—H17	119.9
C4—C5—H10	119.3	C8—C9—H17	119.9
C6—C5—H10	119.3	C9—C10—C11	120.5 (2)
C13—C8—C9	118.89 (19)	C9—C10—H18	119.7
C13—C8—C7	121.02 (17)	C11—C10—H18	119.7
C9—C8—C7	120.09 (18)		

Symmetry codes: (i) −*x*+2, *y*, −*z*+3/2.

### Hydrogen-bond geometry (Å, °)

**Table:**

*D*—H···*A*	*D*—H	H···*A*	*D*···*A*	*D*—H···*A*
O2—H3···N1	0.84	1.87	2.611 (2)	147
